# Stem-cell-derived extracellular vesicles in neurodegeneration and neuroaging: therapeutic potential and challenges

**DOI:** 10.20517/evcna.2025.65

**Published:** 2025-09-30

**Authors:** Mohit Kumar, Sudipta Ray, Susmita Sil

**Affiliations:** Department of Pharmacology and Experimental Neuroscience, College of Medicine, University of Nebraska Medical Center, Omaha, NE 68198, USA.

**Keywords:** Brain aging, neurodegeneration, extracellular vesicles, stem cell therapy

## Abstract

Neuroaging is a complex biological process in which the brain undergoes progressive functional decline marked by synaptic loss, neuroinflammation, and cognitive decline. At the molecular and cellular level, aging is driven by multiple interconnected hallmarks, including genomic instability, telomere attrition, epigenetic alterations, mitochondrial dysfunction, cellular senescence, stem cell exhaustion, and altered intercellular communication. Among these, cellular senescence, a state of irreversible cell cycle arrest, has emerged as a critical contributor to brain aging. Senescent cells accumulate with age, driven by the p53-p21 and p16-pRb pathways, and secrete pro-inflammatory factors via senescence-associated secretory phenotype (SASP), thereby exacerbating neurodegeneration, vascular dysfunction, and cognitive decline. Extracellular vesicles (EVs) are natural nanocarriers of proteins, lipids, and nucleic acids, and have emerged as key mediators of intercellular communication and therapeutics for aging and age-related conditions. EVs derived from various cell types, such as mesenchymal stem cells (MSCs), neural stem cells (NSCs), and induced pluripotent stem cells (iPSCs), can modulate senescence-related pathways, reduce inflammation, and promote tissue repair. Preclinical studies demonstrate that stem-cell-derived EVs can improve cognitive performance, enhance neurogenesis, reduce senescence phenotype, improve neuronal survival through neuroprotective miRNAs (miR-181a-2-3p), suppress neuroinflammation via inhibition of NLRP3 inflammasome, and support synaptic plasticity. Stem cell EVs possess natural biocompatibility, the ability to cross the blood-brain barrier (BBB), and targeted delivery mechanisms, making them promising candidates for anti-aging interventions. This review elaborates on the multifaceted role of stem cell EVs in mitigating brain aging, senescence, and age-associated chronic disease phenotype.

## INTRODUCTION

Aging, broadly defined as organismal/chronological aging, is a complex, time-dependent biological process^[1]^ characterized by gradual loss of tissue homeostasis, metabolic dysfunction, extracellular matrix alterations, chronic inflammation, stem cell depletion, and cellular senescence^[2]^. At the cellular level, aging reflects underlying telomere attrition, mitochondrial dysfunction, oxidative stress, and epigenetic alterations^[3]^. For instance, telomeres, the protective caps on chromosomes, progressively shorten with each cell division due to the "end-replication problem" and insufficient telomerase activity. This shortening triggers replicative arrest (a key mechanism of cellular senescence, even in young organisms under stress) and compromises genomic integrity^[4,5]^. Simultaneously, mitochondrial efficiency declines with age, reducing ATP production and increasing the generation of reactive oxygen species (ROS). This elevated ROS creates oxidative stress, which further damages mitochondria, establishing a self-amplifying vicious cycle of mitochondrial dysfunction and oxidative stress^[6]^. Furthermore, aging involves epigenetic dysregulation such as alterations in chromatin remodeling, histone modifications, DNA methylation, and noncoding RNA expression, which disrupts the precise control of gene expression essential for cell function and overall cellular health^[7]^. Collectively, the cumulative damage from telomere shortening, mitochondrial/oxidative stress, and epigenetic drift drives cellular senescence, a state of irreversible cell cycle arrest^[4,8-11]^, characterized by multiple hallmarks such as increased expression of p16, p21, lysosomal mass, multinucleation, senescence-associated heterochromatic foci (SAHFs), persistent DNA damage foci (e.g., γH2AX), lamin B1 loss, lipofuscin accumulation, and a pathogenic senescence-associated secretory phenotype (SASP)^[12]^. Although cellular senescence contributes to organismal/chronological aging (natural, time-dependent functional decline observed in older individuals)^[13,14]^, it can also be induced independently of chronological age in different contexts, such as stress-induced premature senescence (triggered by factors such as oxidative stress, DNA damage, or metabolic dysfunction)^[15]^, oncogene-induced senescence (aberrant oncogene activation that serves as a tumor-suppressive mechanism)^[16]^, and therapy- or injury-induced senescence (develops in response to radiation, chemotherapeutics, or tissue damage)^[17,18]^. Over time, senescent cell accumulation across organs such as the brain, heart, and kidneys promotes the onset and progression of age-related chronic diseases, including Alzheimer’s disease (AD) and Parkinson’s disease (PD)^[19-22]^. Therefore, it is essential to distinguish between organismal aging, cellular senescence, and age-associated diseases (which are more prevalent in older individuals but are not exclusive to aging itself)^[23,24]^.

In the brain, aging manifests as neuroaging**,** a gradual structural and functional decline marked by synaptic loss, reduced neurogenesis, and impaired homeostasis, leading to mild cognitive impairment (MCI)**,** a preclinical stage of cognitive decay^[25-27]^. Within the brain, a complex network of glial cells and non-neuronal cells exhibits a classical senescence phenotype, contributing to neurodegeneration^[19,28-31]^. Emerging data demonstrate that the accumulation of senescent cells in the central nervous system (CNS) directly contributes to neurodegenerative diseases, and vice versa, by promoting inflammation, disrupting neurovascular units, and impairing synaptic communication^[32-36]^. Critically, post-mitotic neurons, once considered senescence-resistant, have been shown to demonstrate a distinct senescence phenotype in response to aging or injury^[37,38]^. A recent study reported that impaired autophagy in microglia promotes cellular senescence, leading to the emergence of SASP and exacerbation of AD neuropathology in a mouse model^[39]^. Furthermore, in the PD mouse model, nuclear inclusions containing α-synuclein (α-syn) species have been shown to induce transcriptional alterations involving an increase in p21 expression and additional genes related to SASP. These transcriptional changes are linked to DNA damage, lysosomal dysfunction, oxidative stress, chronic inflammation, and gliosis, ultimately accelerating neuronal loss and neurodegeneration^[40]^. Thus, cellular senescence represents not just a biomarker but a mechanistic driver of brain aging and neurodegeneration.

Extracellular vesicles (EVs) are heterogeneous lipid-bilayer particles released by all cells and serve as critical mediators of intercellular communication by transferring proteins, nucleic acids, and lipids between cells^[41]^. EVs are classified into three primary subtypes based on biogenesis pathway and diameter: (1) Exosomes (30-150 nm), formed through the endosomal pathway via inward budding of multivesicular bodies (MVBs) and released upon MVB-plasma membrane fusion, enriched in tetraspanins (CD63, CD81) and endosomal sorting complex proteins (ALIX, TSG101); (2) Microvesicles (100-500 nm), generated by direct outward budding and fission of the plasma membrane, carrying surface phosphatidylserine and selectins; and (3) Apoptotic bodies (500-5,000 nm), produced during programmed cell death through plasma membrane blebbing, containing nuclear fragments and histones^[42-44]^. In the context of senescence and neurodegeneration, EVs play a dual role involving propagating pathology and offering therapeutic promise^[45]^. During aging or pathological insult, neurons and glial cells release EVs enriched with SASP^[30,46-48]^, neurotoxic protein aggregates (such as prion and mutant huntingtin protein)^[49,50]^, and regulatory microRNAs (e.g., G3BP1)^[51]^, ultimately accelerating neurodegenerative processes. Conversely, EVs have emerged as promising diagnostic and therapeutic tools^[52]^. Plasma and brain-derived EVs circulating in biofluids contain disease-specific biomarkers, such as Aβ, p-Tau217, and p-Tau181 for AD^[53-55]^, oligomeric α-syn for PD^[56]^, and TDP-43 in amyotrophic lateral sclerosis (ALS) and frontotemporal dementia (FTD)^[57]^, enabling non-invasive disease monitoring. Moreover, engineered EVs can deliver therapeutic cargoes, including siRNAs targeting mutant mRNA^[58]^, miRNA targeting senescent cells^[59,60]^, neurotrophic factors^[61]^, or even functional mitochondria^[62]^, thereby rejuvenating aged or damaged neural cells^[63,64]^. Recent evidence supports the promising role of stem cell-derived EVs in counteracting senescence and age-related phenotypes involving multiple brain-related diseases and disorders^[65-67]^. Therefore, this review emphasizes the stem cell-derived EVs’ influence on brain aging, senescence, and age-associated brain diseases, and how they can be leveraged to restore brain homeostasis and cognitive function.

## EVs-BASED THERAPEUTICS FOR BRAIN AGING

Neuroaging is mediated by interconnected biological phenomena, including three main pathophysiological mechanisms: chronic neuroinflammation, synaptic dysfunction, and impaired neurogenesis^[25]^. These mechanisms collectively undermine cognitive resilience and increase susceptibility to neurodegeneration, synergistically driving brain aging^[25,68]^. Previous studies have demonstrated that aging is accompanied by brain atrophy, synaptic loss, degeneration of white matter and myelin, hippocampal shrinkage, and progressive cognitive deficits^[69-74]^. Notably, a recent study demonstrated that EVs derived from antler blastema progenitor cells [EV^ABPC^: skeletal mesenchymal stem cells (MSCs) found in regenerating deer antlers] improved brain function in aged models. Specially, administration of these EVs^ABPC^ in aged C57BL/6J mice (18 months old**)** via tail vein injection (40 µg, 3 times weekly for 4 weeks) and to aged rhesus macaques (16-18 years old) via intravenous injection (15 mg, every two weeks for 20 weeks) significantly enhanced physical and cognitive performance, reduced microlgial activation, and reversed epigenetic age by over 3 months in aged mice and more than 2 years^[75]^. Likewise, in aged male Wistar rats (12-13 months old), a single stereotactic injection of adipose tissue stem cell-derived extracellular vesicles (ADSC-EVs; 40 or 100 μg per rat for either 3 or 7 days) conjugated with Exo-pep-11, into the lateral ventricle significantly enhanced neural stem cell (NSC) (cells capable of differentiation into neurons, astrocytes, and oligodendrocytes) self-renewal. Mechanistically, Exo-pep-11 targets EphA4, a receptor tyrosine kinase that normally upregulates phosphatase and tension homolog (PTEN) to suppress protein kinase B (PKB/AKT) phosphorylation, thereby limiting NSC proliferation^[76]^. Blocking EphA4 signaling reduced PTEN expression and activated AKT signaling, which promoted NSC proliferation, survival, and self-renewal^[77]^. These effects are marked by an increase in NSC proliferation and elevated expression of Nestin (an intermediate filament protein essential for NSC cytoskeletal integrity and self-renewal) and DNA-binding protein ID1 (a transcriptional regulator that maintains NSC self-renewal by inhibiting differentiation). Furthermore, these EVs also promotes neurogenesis (the generation of new neurons from NSCs) in the subventricular zone-olfactory bulb axis in these rats, as evidenced by increases in tyrosine hydroxylase (TH**:** a key enzyme in dopamine synthesis and a marker of dopaminergic neuron differentiation) and Tuj1, a neuron-specific class III β-tubulin that serves as an early marker of neuronal maturation and axonal development^[78-82]^. Moreover, cortical injury is known to induce neuronal damage and synaptic loss, mediating significant cognitive and behavioral impairments^[83]^. Aging independently drives progressive cortical thinning through synaptic attrition and reduced neuroplasticity^[84]^. These pathologies differentially manifest across neurodegenerative conditions such as AD and PD^[85,86]^. In 16 to 26-year-old Rhesus macaques, cortical brain injury (induced by surgical procedure) was associated with the loss of excitatory synapses and the presence of the C1q protein, a complement pathway protein critical for microglia-mediated phagocytosis, in perilesional areas^[87,88]^. In contrast, intravenous administration of MSC-EVs (4 × 10^11^ particles/kg at 24 h and 14 days post-injury) has been shown to increase the expression of C1q+ hypertrophic microglia, which were thought to play a role in the clearance of debris and possess anti-inflammatory functions in the perilesional M1 area. Conversely, in the premotor cortices (PMCs), MSCs-EVs treatment is associated with decreased C1q+ synaptic tagging and reduced microglial-spine contacts, facilitating synaptic recovery^[87]^. In the same monkey model, MSC-EV treatment has also been linked to a decrease in microgliosis, enhanced neuronal myelin maintenance, a reduction in the density of damaged oligodendrocytes, and reduced neuronal pathology. These changes correlate with the rate of motor recovery and enhanced synaptic plasticity^[89-91]^.

## EV-BASED THERAPEUTICS FOR CELLULAR SENESCENCE-ASSOCIATED NEURODEGENERATION

While neurodegeneration and cognitive decline are hallmarks of organismal aging, they are also mirrored by cellular senescence, a phenomenon that can occur independently of age in response to stress, injury, or genetic predisposition^[92,93]^. For example, neurological insults such as stroke (significant interruption in cerebral blood flow that leads to severe focal brain injuries), chronic hypoxia/ischemia (a sustained reduction in oxygen and nutrient supply that disrupts neuronal metabolism and accelerates neurodegeneration), metabolic stress (e.g., insulin resistance), and neurodegenerative diseases accelerate senescence across multiple neural populations, including NSCs, neurons, and microglia^[94-97]^. Senescent neural cells not only lose their functional potential but also promote neurodegeneration through the SASP^[98]^. Recent evidence highlights the therapeutic potential of EVs derived from various stem cell sources in mitigating neural senescence and restoring normal functioning. For instance, in an induced neural stem cell (iNSC) model of ischemic stroke, EVs from hypoxic-preconditioned human embryonic stem cells (hESCs-HypoxEVs, 40 ug/mL for 24 h), enriched with glutathione (GSH) redox system, effectively attenuated cellular senescence by significantly reducing expression of the senescence markers p16 and SA-β-Gal, while also suppressing key SASP factors including IL-6, CXCL8, and MMP9^[95]^. Complementing these findings, a study using primary NSCs from neonatal C57BL/6J mice demonstrated that NSCs subjected to oxygen-glucose deprivation (OGD) undergo senescence, leading to a decline in both NSC differentiation and neurogenesis^[63]^. Likewise, in an *in vitro* insulin-resistant neural stem and progenitor cell (NSPC) model, insulin and palmitic acid (IPA) induced senescence characterized by upregulation of p16^INK4a^ and p21, and impairment of IRS-1/Forkhead box O (FoxO) signaling, which reduced FoxO1/3a recruitment to proliferation-related genes such as Cyclin D and SRY-box transcription factor 2 (SOX2). In contrast, treatment of cultured NSPCs with NSPC-derived exosomes (2.5 μg of vesicles per 1 × 10^6^ cells) for 3 days significantly mitigated IPA-induced alterations and reversed proliferation deficits, thereby counteracting the senescent phenotype^[96]^. Furthermore, in a D-galactose-induced BV2 microglial senescence model, induced pluripotent stem cell (iPSC)-derived EVs (iPSC-EVs, 1 × 10^9^ particles/mL) significantly reduced senescence markers (p16, p21, p53, γ-H2AX, and SA-β-Gal) and attenuated classically activated M1 microglia pro-inflammatory polarization, while promoting an anti-inflammatory phenotype. Mechanistically, these EVs deliver TGF-β1, which upregulates Rictor and activates AKT phosphorylation, thereby suppressing both inflammation and senescence^[93]^. Moreover, *in vivo* studies in senescence-accelerated mouse prone 8 (SAMP8) and the C57BL/6 mouse models of varying ages identified age-related senescence in hippocampal NSCs (H-NSCs), linked to reduced hippocampal neurogenesis, a critical process for maintaining and restoring hippocampus-dependent cognitive functions. This reduction contributes to cognitive deficits often observed in aging and neurodegenerative disorders. In contrast, intravenous administration of ESCs-EVs counteracts H-NSCs senescence in both SAMP8 (6-month treatment) and C57BL/6 (8-month treatment) mice. This effect is mediated through the upregulation of myelin transcription factor 1 (MYT1), which activates downstream signaling pathways involving SIRT1, nicotinamide phosphoribosyl transferase (NAMPT), and hypoxia-inducible factor 2 subunit α (HIF-2α)^[99-101]^. Additionally, MSC-derived exosomes (MSCs-Exos) have been shown to reduce oxidative stress, exert anti-apoptotic effects, and increase SIRT1 levels, thereby preventing brain aging in SAMP8 mice^[66]^. Mechanistically, SIRT1, NAMPT, and HIF-2α form a coordinated axis that regulates neurogenesis by linking metabolic and hypoxic signaling. NAMPT maintains NAD+ levels, enabling SIRT1 activation, which deacetylates HIF-2α to enhance its stability and transcriptional activity, thereby promoting NSC survival, differentiation, and vascular support^[102-105]^.

## EV-BASED THERAPEUTICS FOR AGE-RELATED CHRONIC DISEASES

Age-related chronic diseases, including AD and PD, result from progressive cellular and molecular impairments^[106,107]^. These impairments overlap with those of brain aging, thereby accelerating cognitive decline and neurodegeneration^[108-111]^. Emerging evidence highlights stem cell-derived EVs as a promising therapeutic agent, capable of delivering bioactive molecules that restore neural homeostasis and attenuate disease progression. Understanding these mechanisms is essential for developing targeted strategies to preserve brain health during aging.

## EV-BASED THERAPEUTICS FOR AD

AD represents a prime example of accelerated neuroaging, pathologically characterized by the accumulation of Aβ plaques and neurofibrillary tangles composed of hyperphosphorylated tau protein^[41,112]^. Epidemiological studies demonstrate that the risk of developing AD doubles every decade after age 65^[113]^. Mounting evidence indicates senescent cell accumulation in the brain of AD patients^[114-116]^, suggesting their potential role in disease progression. Supporting this, pharmacological clearance of senescent cells in AD mouse models has been shown to reduce Aβ deposition, tau pathology, and improve cognitive function^[117-120]^. In parallel, recent studies demonstrated that exposing human iPSC-derived neurons to human iPSC-NSC-EVs (6 × 10^9^ EVs) effectively counteracts Aβ42-induced neurotoxicity through multifaceted mechanisms, including antioxidant, anti-apoptotic, pro-autophagic, and anti-tauopathy effects, positioning them as a promising cell-free therapeutic approach for AD^[97]^. Likewise, mesenchymal stem cell-derived EVs enriched with tyrosine phosphatase-2 (SHP2) (MSCs-EVs-SHP2) have been shown to induce mitophagy in SH-SY5Y neuronal cells *in vitro* and an experimental model of AD [intracerebroventricular injection of Aβ_1-42_ (5 mg/mL)] with a treatment regimen of 100 µg of EVs per mouse every two days, for 2 weeks. These EVs enhanced mitochondrial function, reduced NLRP3 inflammasome activation, and decreased neuronal apoptosis in the cerebral cortex, ultimately rescuing synaptic loss and improving cognitive function^[121]^. Additionally, both MSCs-EVs and iNSCs-EVs exhibit anti-aging effects by mitigating AD-like phenotypes. In four-month-old 5XFAD mice, intravenous administration of MSCs-EVs and iNSCs-EVs (0.5 μg/μL, every three days for one month) has been shown to reduce amyloid plaques, inflammatory responses, and improve dendritic spine density and arborization in the prefrontal cortex and hippocampus, thereby enhancing learning and memory functions^[122]^. In another study using two- or six-month-old 5XFAD mice, intravenous (retro-orbital vein) administration of hNSCs-EVs (once or twice over 4-6 weeks) has been shown to reduce microglial activation, lower pro-inflammatory cytokine levels, decrease amyloid-beta plaque accumulation, mitigate synaptic loss, and improve cognitive function^[123]^. Similarly, in 3-month-old 5xFAD mice, intranasally delivered hiPSCs-NSCs-EVs (30 × 10^9^ EVs, once weekly for 2 weeks) were internalized by astrocytes and microglia, surrounding the plaques. Moreover, these EVs diminish microglia activation by downregulating genes associated with NOD-, LRR-, and pyrin domain-containing protein 3 (NLRP3) inflammasome components and IFN-1 signaling pathways. Concurrently, these EVs inhibit Interleukin 6 (IL-6) and IFN-1 signaling in astrocytes. Overall, EV therapy reduced hippocampal amyloid-beta plaques, p-tau accumulation, astrocyte hypertrophy, and microglial clustering, hence enhancing cognitive functions^[124]^.

## EV-BASED THERAPEUTICS FOR PD

**Figure 1:**
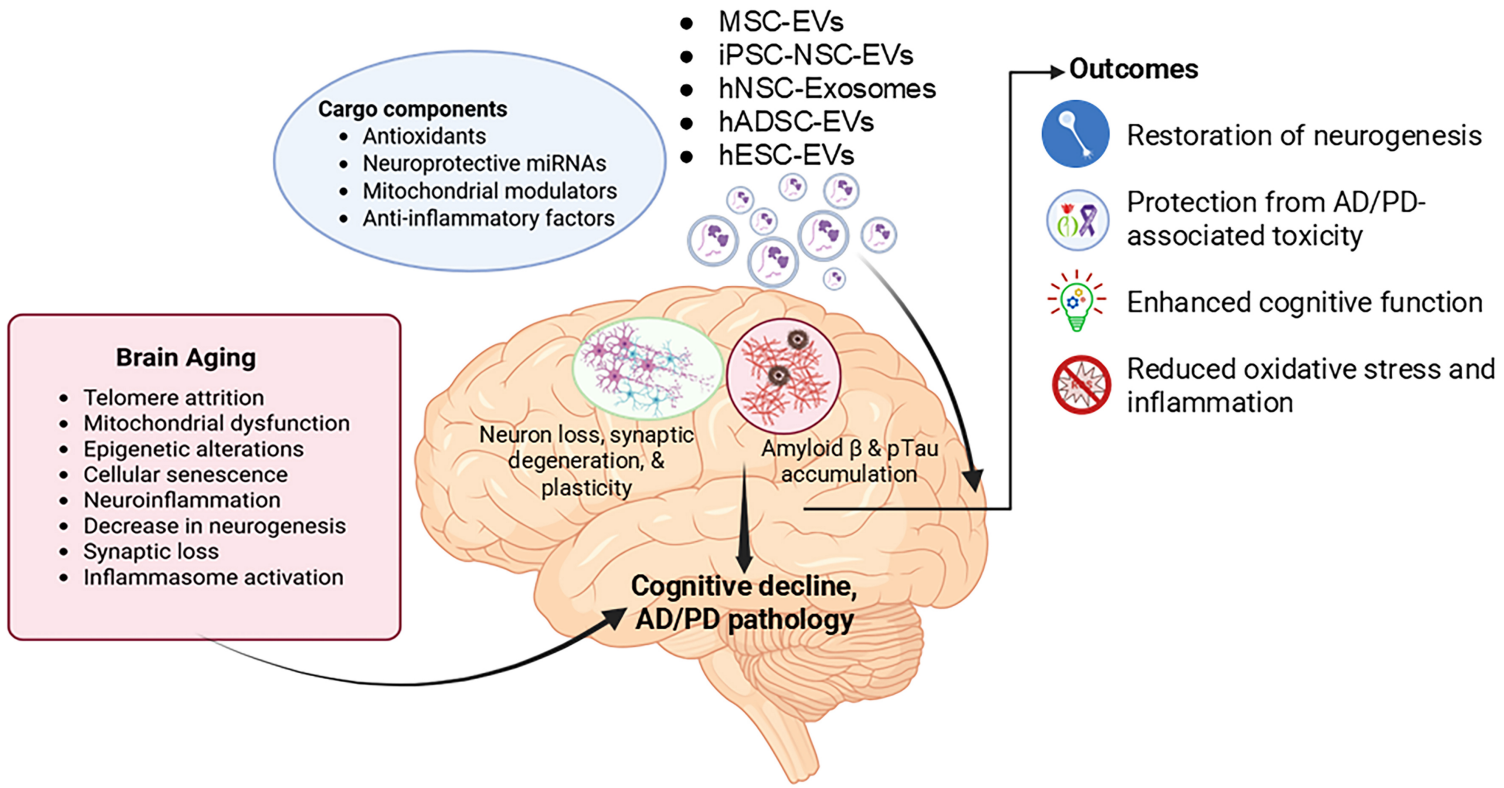
Schematic representation of therapeutic intervention by stem cell-derived EVs in Aging. Aging triggers cellular senescence, neuroinflammation, and synaptic dysfunction, driving cognitive decline and contributing to Alzheimer’s and Parkinson’s disease pathology. Stem cell-derived extracellular vesicles carry antioxidants, neuroprotective microRNAs, mitochondrial regulators, and anti-inflammatory molecules. These vesicles enhance neurogenesis, protect against neurotoxicity, alleviate oxidative stress and inflammation, and improve cognitive function. (Created in BioRender. https://BioRender.com/u7czgbc). EVs: Extracellular vesicles; MSC: mesenchymal stem cell; iPSC-NSCs: induced pluripotent stem cell-derived neural stem cells; hNSCs: human neural stem cells; hADSCs: human adipose-derived stem cells; AD: Alzheimer’s disease; PD: Parkinson’s disease.

PD is another age-related neurodegenerative disorder characterized by the progressive loss of dopaminergic neurons in the substantia nigra (SN) and accumulation of α-syn protein aggregates, driven by interconnected cellular pathologies including mitochondrial dysfunction, impaired protein clearance, chronic neuroinflammation, and selective neuronal vulnerability to oxidative stress and calcium dysregulation^[125,126]^. This degenerative cascade is initiated by the downregulation of key regulatory molecules such as the neuroprotective proteins (Parkin), the synaptic regulator [DJ-1, also known as Parkinson’s disease protein 7 (PARK7)], and dysregulated microRNAs (including miR-181a-2-3p)^[127-129]^. miR-181a-2-3p, which plays a key role in regulating autophagy and apoptosis, has been consistently found to be downregulated in PD, both in patient tissues and experimental animal models^[129,130]^. This downregulation has been further confirmed in SH-SY5Y cells and PD mouse models following exposure to 6-hydroxydopamine (6-OHDA), a neurotoxin commonly used to stimulate PD pathology^[131]^. However, treatment with MSC-EVs (200 µg EV-protein, every 3 days for 8 weeks) effectively restored miR-181a-2-3p expression in both SH-SY5Y cells and mouse brain tissue (administered via tail vein). Restoration of this microRNA led to enhanced neuronal viability and antioxidant defenses, marked by increased superoxide dismutase (SOD) levels, and concurrently reduced apoptosis and oxidative stress indicators such as ROS and malondialdehyde (MDA). Mechanistically, miR-181a-2-3p directly targets and suppresses early growth response 1 (EGR1), a transcription factor that upregulates NOX4, a major driver of oxidative stress through activation of the p38 MAPK pathway, thus alleviating oxidative damage and supporting neuronal cell survival^[131]^. Furthermore, in the same mouse model, administration of human umbilical cord MSC-derived exosomes (hucMSCs-Exos; 200 µg EV protein, every 3 days for 8 weeks, tail vein injection) has been demonstrated to reach the SN, significantly reduce dopaminergic neuronal loss, and upregulate dopamine levels and its metabolites^[132]^. Likewise, in the rotenone-induced rat model of PD, repeated tail vein administration of neural-induced human adipose tissue-derived stem cell exosomes (NI-hADSC-Exos, 500 µg/kg of EV protein) significantly attenuated neurodegeneration, leading to improved motor function and enhanced survival of dopaminergic neurons. The treatment reduced levels of pathogenic oligomeric phosphorylated α-synuclein (p-S129 α-syn) and suppressed astrocytic and microglial activation. Mechanistically, NI-hADSC-Exos restored autophagic flux by downregulating dysregulated serine/threonine protein kinases, thereby promoting α-syn clearance and inhibiting mitochondria-mediated apoptosis^[133]^. Thus, the above-mentioned findings highlight the potential of stem cell-derived EVs in counteracting brain aging, mitigating cellular senescence, and alleviating age-related chronic diseases, while simultaneously promoting neuroprotection. These properties position EVs as promising candidates for treating neurodegenerative disorders and advancing regenerative medicine [Figure 1 and Table 1].

**Table 1:** Therapeutic effects of stem cell-derived extracellular vesicles on aging and neurodegenerative disorders

**Stem cell type**	**EV source**	**Administration**	**Mechanism/Target**	**Disease model**	**Reference**
Antler blastema stem cells	Antler pedicle periosteum and regenerating antler tissue of sika deer	Tail vein injection	Intravenous EVs improved physical performance, enhanced cognitive function, and reduced systemic inflammation in aged mice, while reversing epigenetic age by over 3 months. In macaques, EV treatment was also neuroprotective, reduced inflammation, improved locomotor function, and reduced epigenetic age by over 2 years	Aged macaques and mice	Hao *et al*. (2025)^[75]^
Mesenchymal stem cells	Bone marrow-derived MSCs of a single young monkey	Intravenous	EV treatment promoted debris clearing, anti-inflammatory C1q+ microglia in the perilesional motor cortex, aiding recovery. In premotor regions, EVs reduced C1q+ synaptic tagging and microglia-spine contacts, suggesting improved synaptic plasticity through local damage clearance and prevention of chronic inflammation	Aged rhesus macaques with cortical injury	Zhou *et al*. (2023)^[87]^
Mesenchymal stem cells	Bone marrow-derived MSCs of a single young monkey	Intravenous	EV treatment reduced oligodendrocyte damage and enhanced myelin maintenance in sublesional white matter. These effects correlated with motor recovery, suggesting EVs promote repair and functional improvement in the aged brain	Aged rhesus macaques	Go *et al*. (2021)^[89]^
hESCs	Hypoxia-conditioned hESC line BG01 (WiCell Research Institute)	Intravenous injection	hESC-EVs reversed neural stem cell senescence after ischemic injury by suppressing SASP factors and restoring neuroprotection, with hypoxia-preconditioned EVs showing enhanced GSH-dependent anti-senescence activity. MFGE-8-coated hESC-HypoxEVs further promoted neurogenesis and improved sensorimotor recovery	MCAO model from eight-week-old male SD rats	Lee *et al*. (2025)^[95]^
Postnatal hippocampal NSPC	Newborn (0-1 days) C57bl/6 mice	Intranasal administration of exosomes from NSPC	NSPC-derived EVs reverse insulin resistance-induced senescence by restoring IRS-1/FoxO signaling, reducing p21, and preserving hippocampal neurogenesis	Thirty- to 35-day old C57BL/6 mice	Natale *et al*. (2022)^[96]^
Human ESC-sEVs	Human ESC (H9) provided by the Institute of Biochemistry and Cell Biology of the Chinese Academy of Sciences (Shanghai, China)	Intravenous injection	ESC-sEVs rejuvenate aging hippocampal NSCs by transferring SMAD4/5 to activate the MYT1-Egln3-SIRT1 axis, restoring neurogenesis and cognition	SAMP8 & C57BL/6 mice	Hu *et al*. (2021)^[99]^
MSC-Exos	Mouse BM-MSCs	Tail vein injection	MSC-derived exosomes delay brain aging by activating SIRT1, reducing oxidative stress and apoptosis, and improving cognition	SAMP8 senescent mice	Zhang *et al*. (2023)^[66]^
hiPSC-NSC-EVs	hiPSC-NSC	*In vitro*	hiPSC-NSC-EVs protect neurons from Aβ-42-induced senescence-like changes by reducing oxidative stress, restoring mitochondrial function, and suppressing tau pathology	Mature human neurons differentiated from human NSCs	Rao *et al*. (2025)^[97]^
Mesenchymal stem cells	Engineered mesenchymal stem cell-derived extracellular vesicles with tyrosine phosphatase-2 (SHP2) high expression (MSC-EVs-SHP2)	Intravenous injection	SHP2-enriched MSC-EVs induce mitophagy, reducing mitochondrial damage, neuroinflammation, and cognitive decline in the AD mice model	AD was established in C57BL/6 mice by injecting 5 mg/mL^-1^ β-amyloid (1-42) into the lateral ventricle of the cerebrum	Xu *et al*. (2022)^[121]^
Mouse NSCs and iNSCs	Conditioned medium of cultured mouse NSCs and iNSCs	intravenous injection	NSC-EVs mitigate AD-related aging phenotypes by reducing amyloid and inflammation, restoring synapses, and improving cognition	5XFAD mice	Gao *et al*. (2023)^[122]^
hNSC	EVs from conditioned medium of cultured hNSCs	RO sinus injection	hNSC-EVs mitigate Alzheimer’s hallmarks by reducing amyloid and tau pathology, restoring synapses, and improving cognition	5XFAD mice	Apodaca *et al*. (2021)^[123]^
Mesenchymal stem cells	EVs from hucMSCs	Tail vein injection	MSC-EVs deliver miR-181a-2-3p to inhibit the EGR1/NOX4 pathway, reducing oxidative stress and protecting dopaminergic neurons in PD	6-OHDA-induced PD model (SH-SY5Y cells and C57BL/6 mice)	Ma *et al*. (2022)^[131]^
Mesenchymal stem cells	EVs from hucMSCs	Intravenous injection	hucMSC-Exos induce autophagy, protect dopaminergic neurons, restore dopamine, and improve motor function in PD models.	6-OHDA-induced PD model (SH-SY5Y cells and adult male SD rats)	Chen *et al*. (2020)^[132]^
NI-hADSC	NI-hADSC-Exo from adipose tissues of human donors	Intravenous injection	NI-hADSC-Exos clear α-syn aggregates, restore autophagy, reduce inflammation, and protect dopaminergic neurons in PD rats	Rotenone (subcutaneous)-induced Parkinson’s disease rat model	Ramalingam *et al*. (2025)^[133]^

EV: Extracellular vesicle; MSC: mesenchymal stem cell; hESC: human embryonic stem cell; hESC-EV: human embryonic stem cell-derived extracellular vesicle; hESC-HypoxEV: hypoxia-conditioned human embryonic stem cell-derived extracellular vesicle; MFGE-8: milk fat globule-EGF factor 8; GSH: glutathione; SASP: senescence-associated secretory phenotype; MCAO: middle cerebral artery occlusion; SD: Sprague Dawley; NSPC: neural stem and progenitor cell; IRS-1: insulin receptor substrate-1; FoxO: forkhead box O; ESC: embryonic stem cell; sEV: small extracellular vesicle; ESC-sEV: embryonic stem cell-derived small extracellular vesicle; NSC: neural stem cell; hiPSC: human induced pluripotent stem cell; hiPSC-NSC: hiPSC-derived neural stem cell; hiPSC-NSC-EV: hiPSC-NSC-derived extracellular vesicle; Aβ-42: amyloid beta-42; SHP2: Src homology region 2-containing protein tyrosine phosphatase-2; AD: Alzheimer’s disease; 5XFAD: transgenic Alzheimer’s disease mouse model; RO: retro-orbital; hNSC: human neural stem cell; hucMSC: human umbilical cord mesenchymal stem cell; hucMSC-Exo: hucMSC-derived exosome; miR-181a-2-3p: microRNA-181a-2-3p; EGR1: early growth response 1; NOX4: NADPH oxidase 4; PD: Parkinson’s disease; 6-OHDA: 6-hydroxydopamine; SH-SY5Y: human neuroblastoma cell line SH-SY5Y; NI-hADSC: neural-induced human adipose tissue-derived stem cell; NI-hADSC-Exo: NI-hADSC-derived exosome; α-syn: alpha-synuclein; hucMSCs: human umbilical cord mesenchymal stem cells.

## CONCLUSION AND FUTURE PERSPECTIVES

Stem cell-derived EVs have gained increasing interest as a potential non-cellular therapeutic platform to counteract brain aging, cellular senescence, and age-related brain disorders, including AD and PD. These nanosized lipid bilayer vesicles are endogenous carriers of proteins, nucleic acids, lipids, and metabolites, which parallels the molecular signature of their parent cells. In contrast to stem cell transplantations, EV-based therapy avoids risks such as tumorigenicity, immune rejection, and ethical concerns, while retaining the same important properties in tissue repair and immune modulation. As discussed in this article, EVs from MSCs, NSCs, hESCs, and iPSCs have demonstrated significant potential in rejuvenating senescent neural cell populations, promoting neurogenesis, restoring synaptic plasticity, and ameliorating behavioral deficits in diverse models of aging and neurodegeneration^[59,64,75,90]^. For instance, in aged mice and rhesus macaques, EVs isolated from antler blastema progenitor cells significantly reversed epigenetic age, improved cognitive function, and reduced systemic inflammation^[75]^. Additionally, EVs from adipose-derived stem cells conjugated with Exo-pep-11 enhanced neural stem cell proliferation by targeting the EphA4/PTEN/AKT axis, thereby improving self-renewal and neurogenesis^[78]^. Hypoxia-preconditioned hESC-EVs enriched with GSH suppressed senescence markers and SASP in ischemia-induced NSCs^[95]^, while iPSC-derived EVs significantly lowered markers of cellular aging and shifted microglia from a pro-inflammatory (M1) state toward a more anti-inflammatory profile^[93]^. Similar regenerative effects have been observed across PD models, where stem-cell EVs restored dopaminergic neuronal function, reduced α-syn aggregates, and modulated neuroinflammation via microRNA- and mitophagy-based mechanisms^[131-133]^.

Despite these promising results, clinical translation of stem cell-derived EV therapies is limited by a number of unresolved biological and technical barriers. One of the biggest concerns is that of immunocompatibility. While EVs are commonly described as immune-evasive due to the lack of MHC class II molecules, emerging evidence suggests that allogeneic EVs could still carry immune-surface proteins or membrane-bound antigens that could lead to an immune response, especially upon repeated systemic exposure^[134,135]^. Another obstacle is the heterogeneity of EV populations. Even within EVs secreted by a specific cell type, significant heterogeneity can be observed with respect to size, content, and surface markers, as determined by culture conditions, passage number, and EV biogenesis pathway^[136,137]^. This heterogeneity presents challenges to the reproducibility of therapeutic effects, as well as the standardization of manufacturing processes for clinical use. Moreover, the pharmacokinetics of EVs are suboptimal. Stem cell-derived EVs exhibit rapid clearance and poor brain penetration. For example, radiolabeling studies of human umbilical cord MSC-EVs with Zirconium-89 revealed a biphasic circulation pattern with a distribution phase half-life of ~28 min and an elimination phase half-life of ~12 h, with predominant accumulation in the liver and spleen, and < 0.05% of the injected dose reaching the brain^[138]^. Similarly, technetium-99m-labeled hUC-MSC-EVs showed a distribution phase half-life of ~1.13 min and an elimination phase half-life of ~45.98 min, with most vesicles sequestered in the liver and spleen, and only trace amounts (< 0.1%) detected in the brain^[139]^. These findings significantly limit their therapeutic efficacy in neurodegenerative settings.

The targeting of cells and functional delivery also further restricts the scope of therapeutic application of these EVs. The majority of EVs are taken up into cells by non-specific endocytosis, subsequently undergoing lysosomal degradation. Endosomal escape is a significant hurdle due to inefficient cytoplasmic delivery of the therapeutic RNA and protein cargo of the EVs^[140]^. Although still under preclinical investigation, some engineering strategies, such as the use of fusogenic peptides and pH-responsive coatings, are being examined^[141]^. Off-target effects remain another major concern, especially as EVs have been shown to accumulate in peripheral organs. For immunotherapy, tumor-promoting miRNAs or growth factors could, in principle, be passed through pluripotent stem cell-derived EVs^[142]^. These risks emphasize the importance of rigorous preclinical safety assessments and long-term toxicity studies. Additionally, the successful translation of EVs to clinical use will require the scalable and reproducible manufacturing of GMP-grade EVs through well-benchmarked protocols for isolating, characterizing, and testing for potency of EVs^[143]^. Storage conditions, such as cryopreservation and lyophilization, need to be optimized to preserve their bioactivity^[144]^.

In summary, EVs from stem cells represent an exciting new therapeutic platform that has the potential to rejuvenate neural systems, intervene in senescence, and fight neurodegeneration. However, their efficient transfer from laboratory to clinic needs to overcome several barriers, including immunogenicity, heterogeneity, pharmacokinetics, biodistribution, targeting, endosomal escape, and large-scale production. Such challenges will require continued interdisciplinary collaboration across neuroscience, bioengineering, immunology, and regulatory science. With continuous research to overcome these obstacles, EVs derived from stem cells have the potential to emerge as a breakthrough treatment for neurodegenerative disorders and in the promotion of healthy brain aging.

## DECLARATIONS

### Copyright

© The Author(s) 2025.
